# Evaluating YOLO architectures for detecting road killed endangered Brazilian animals

**DOI:** 10.1038/s41598-024-52054-y

**Published:** 2024-01-16

**Authors:** Gabriel Souto Ferrante, Luis Hideo Vasconcelos Nakamura, Sandra Sampaio, Geraldo Pereira Rocha Filho, Rodolfo Ipolito Meneguette

**Affiliations:** 1 Institute of Science Mathematics and Computer Science, University of São Paulo, 400 Trabalhador São-carlense Avenue, São Carlos, São Paulo 13566-590 Brazil; 2 Departament of Informatics, Federal Institute of São Paulo - Campus Catanduva, 239 Pastor José Dutra de Moraes Avenue, Catanduva, São Paulo 15808-305 Brazil; 3 Department of Computer Science, University of Manchester, Oxford Rd, Manchester, M13 9PL UK; 4 Department of Exact and Technological Sciences, State University of Southwest Bahia, Estr. Bem Querer, Km-04, Vitória da Conquista, BA 45083-900 Brazil

**Keywords:** Computer science, Information technology, Scientific data

## Abstract

Wildlife roadkill is a recurring, dangerous problem that affects both humans and animals and has received increasing attention from environmentalists worldwide. Addressing this problem is difficult due to the high investments required in road infrastructure to effectively reduce wildlife vehicle collisions. Despite recent applications of machine learning techniques in low-cost and economically viable detection systems, e.g., for alerting drivers about the presence of animals and collecting statistics on endangered animal species, the success and wide adoption of these systems depend heavily on the availability of data for system training. The lack of training data negatively impacts the feature extraction of machine learning models, which is crucial for successful animal detection and classification. In this paper, we evaluate the performance of several state-of-the-art object detection models on limited data for model training. The selected models are based on the YOLO architecture, which is well-suited for and commonly used in real-time object detection. These include the YoloV4, Scaled-YoloV4, YoloV5, YoloR, YoloX, and YoloV7 models. We focus on Brazilian endangered animal species and use the BRA-Dataset for model training. We also assess the effectiveness of data augmentation and transfer learning techniques in our evaluation. The models are compared using summary metrics such as precision, recall, mAP, and FPS and are qualitatively analyzed considering classic computer vision problems. The results show that the architecture with the best results against false negatives is Scaled-YoloV4, while the best FPS detection score is the nano version of YoloV5.

## Introduction

Human-wildlife conflicts have a long history, stretching from the dawn of civilization to the present day. In recent times, these conflicts have intensified due to a variety of reasons, including climate change, economic and urban development^1–3^. Conflicts on roads worldwide are receiving increasing attention, with numerous reports of roadkill incidents involving victims such as deer, foxes, rabbits, blackbirds, hedgehogs and pheasants, to name a few^4–6^. These reports are driving environmental research focused on wildlife roadkill, primarily aimed at identifying species on the brink of extinction, but also population trends, species distribution, behaviour and spread of diseases^7^.

In Brazil, roadkill incidents have been recorded in all regions of the country, causing deaths and increasing species extinction rates^8^. According to Centro Brasileiro de Ecologia em Estradas ^9^, about 475 million animals die on brazilian roads each year, including a wide range of species, with small-sized animals accounting for 90% of the victims, followed by medium-sized animals (9%) and large-sized animals (1%). In the State of São Paulo alone, approximately 3,000 collisions involving animals are recorded each year^10^. The main species at risk of extinction are medium and large mammals, such as the Maned Wolf species^11^, Giant Anteaters, Tapirs, Jaguarundis and Pumas^12^. Despite this grim scenario, few roads have been redesigned with roadkill mitigation in mind, such as through the construction of fauna bridges, tunnels and fences around them^13^. In addition, existing technological solutions for automatic animal detection and classification have not been successfully implemented.

Projects such as RoadLab^14^ demonstrate the use of citizen science in collecting data on roadkill incidents involving wildlife. However, computer vision systems are gaining momentum as a data collection mechanism, due to their image classification, feature extraction, object detection and tracking capabilities, among others which involve the use of image processing combined with artificial intelligence. Examples of applications where computer vision technology has been heavily employed include autonomous cars^15^, urban traffic monitoring^16^, car parking management^17^, plate recognition^18^,fault recognition in industrial processes^19^, animal species recognition^20^, and other applications where image-based pattern recognition is necessary.

In particular, computer vision has played a key role in animal classification and detection tasks, aiding in the monitoring of endangered species and fauna identification in green spaces^21^. Additionally, as shown in the work by^22–25^, computer vision has been used in previous efforts to mitigate disruption of natural habitats caused by illegal hunting and highway roadkill, as well as animal invasion of urban areas, farms and plantations.

Computer vision systems typically use machine learning, such as extation of Convolutional Neural Networks (CNN)^26^, to perform object detection and classification. This can be done either as a single-stage process using *single-stage* detectors or as a multi-stage one using multi-stage detectors. Single-stage detectors have been shown to be the most suitable mechanism for real-time object detection^27^ due to their low inference times and *frames per second* rates in relation to accuracy^28^. Among existing single-stage detectors, those with a YOLO-based architecture (*You Only Look Once*)^29^ stand out in performance under benchmarks such as MS COCO (MS COCO is a reference database for benchmarks on object detection.). Additionally, their successful application in animal detection and classification has gained widespread recognition^30,31^, resulting in the emergence of numerous variations of these detectors over the years, making selection of a detector for specific training a complex task. This complexity is particularly evident in scenarios where Edge Computing devices are used in object detection and where considerations around limitations in processing power and other resources need to be made.

With a multitude of alternatives, successful detector selection can be achieved through performance evaluation of the different architecture and model variants, using training based on target-domain datasets. However, availability of consolidated datasets is not guaranteed and the use of small-sized datasets is likely to result in unreliable models, prone to overfitting.

This work addresses the challenge of automatically detecting and classifying road-killed animals using computer vision technology, while taking into account limitations in the availability of target-domain training datasets as well as difficulties associated with animal detection and classification. The goal is to generate statistics about the animal species that are most commonly killed on roads. A comprehensive performance evaluation of state-of-the-art YOLO-based detectors is carried out to identify the most suitable detectors for this task. The evaluation aims to demonstrate the effectiveness of these detectors in training high-precision and high-recall models using small target-domain datasets. To overcome limitations in data availability, transfer learning and data augmentation techniques are employed. The evaluated detectors based on YoloV architecture are described in the paper, include YoloV4 Darknet, Scaled-YOLOv4, YoloV5, YoloR, YoloX and YoloV7. The BRA-Dataset^32^, which contains five classes of animals commonly killed on brazilian roads, is used to validate the detection models. The models are compared using the metrics of mAP@50, precision and recall, as well as their average rate of Frames Per Second (FPS) on a set of web-available animal video recordings and a video recorded at the Ecological Park of São Carlos in Brazil. Incrementally, the detectors are also evaluated in performance over an edge computing device with limited computational resources. The main contributions are summarized as follows:

- A detailed account of the evolution of existing detectors considering real-world applications, including animal detection.
- A comprehensive evaluation of state-of-the-art detectors using several quantitative and qualitative metrics, including image quality aspects related to common challenges in animal detection on highways/roads, such as animals in occluded or challenging positions (e.g., small animals far away from the camera), presence of surrounding vegetation, and low-quality images that can hinder animal detection (e.g., shadowed and not-fully visible animals). The metrics and qualitative aspects considered in this paper go beyond those typically found in other studies involving animal detection/classification.
- A quantitative and qualitative analysis of results, providing valuable insights.
- An indication of the suitability of different detection models for deployment on edge or mobile devices with limited resources, considering their performance properties and complexity.

This paper is organized into six sections. Section “Related works” details the main related works. Section “YOLO architectures” provides an overview of the selected YOLO architectures. Section “Methodology” describes the methodology chosen for evaluating the models. Section “Results” presents the results obtained and comparisons made. Finally, Section “Conclusion” discusses the results and presents conclusions.

## Related works

In this section, the main works involving comparative architectures of convolutional neural networks for animal detection are presented. Among these, works that deal with more than one model are included. In addition, works that use architectures other than YOLO are also considered (Table 1), allowing for a retrospective analysis of the evolution of detectors in real-world applications involving human-animal conflict or animal detection challenges.

In the Brazilian Pantanal region, ecology researchers face challenges in identifying and measuring species density. These tasks are part of their efforts to combat the degradation of the Pantanal. However, the process they employ is generally slow and costly, requiring several days of movement in the forest and the use of camera traps to capture images of animals in a given area. In the work by de Arruda et al.^26^, the use of CNN was proposed to automatically detect and identify animal species from the Pantanal, with the segmentation of regions of interest in thermal and RGB images. The CNN with VGGNet architecture and the SLIC algorithm were chosen for segmentation, while the classic Fast-RCNN was used for comparison. These networks were tested in a sub-set of ImageNet with animals from the Pantanal (Brazilian Tapir, Blue and Yellow Macaw, Puma, Caititu, Capybara and True Parrot, among others). The results demonstrate that the VGGNet method with SLIC surpassed the accuracy of the Fast-RCNN architecture.

In the work of Schneider et al.^33^, deep neural networks were employed for object detection tasks, such as identifying, counting, and locating animals in images captured by camera traps. The great challenge is that these images can present several variations in the positioning of the animal of interest, such as ambient occlusions, irregular lighting, poses at challenging angles, and cuts of the complete location of the animal, which is challenging for computer vision algorithms. A two-stage model Faster R-CNN is compared with a single-stage model YoloV2. These models are compared in terms of speed for real-time detections and accuracy on two trap image datasets, The Reconyx Camera Trap (RTC) and Snapshot Serengeti. The results showed that the Faster R-CNN model showed better performance in terms of accuracy than the YoloV2 model in both datasets, concluding that this technique is very effective for detections in trap camera image processing.

The work of Biswas et al.^34^ aimed to compare the performance of CNNs in detecting bird species in the city of Bangladesh, India. With over 800 species present in the city, manual classification would be impractical. However, the application of machine learning models made this task possible. Seven species were chosen for training with transfer learning over 2800 images and 700 images for validation and testing on the DenseNet201, InceptionResNetV2, MobileNetV2, ResNet50, ResNet152V2, and Xception models. The results of accuracy, precision, recall and *F1-score* were evaluated. The study shows that the MobileNetV2 and Xception models obtained the highest values of results in all metrics. It is concluded that the MobileNetV2 model is the superior model compared to the others, in addition, it can also be concluded that even with a small set, the model obtained high results for bird recognition.

In another study, Adami et al.^35^ compared CNNs to develop a solution that combines edge and cloud computing with computer vision to safely deter animals such as wild boars and deer from agricultural areas on farms. The computer vision system interacts with the edge module through devices specialized in deep learning processing (Intel Movidius Neural Compute Stick (NCS) and NVIDIA Jetson Nano) coupled to a Raspberry Pi Model 3 B+. The real-time object detectors used are YoloV3 and YoloV3-Tiny (a lighter version of YoloV3). The recall metrics, Average Precision (AP), and Mean Average Precision (mAP) of both models are evaluated. The performance in frames per second (FPS) of the models implemented with and without special devices for neural networks at the edge is also evaluated. According to the experiments, YoloV3 obtained better overall performance with 82.5% of mAP, being superior to its the tiny version reached 62.4% mAP. However, in terms of FPS, the Tiny model together with the Nvidia Jetson obtained the best result, with 15 FPS achieved, in contrast, the YoloV3 model only obtained 4 FPS in its best result (with the Jetson Nano). It is concluded that the type of network influences the performance of detections in edge computing, even in soft-real-time tasks.

For the identification and detection of herds of white rhinos, giraffes, wildebeests, and zebras, the work of Petso et al.^30^ used YOLO-based detectors for detection through images captured by drones. The challenge is to detect animals with aerial images, as there may be animals camouflaged with the environment. In addition, the main objective of the implementation was to create a monitoring mechanism with computer vision to keep rhinos safe from the risk of hunting. For this, a dataset with aerial images with images at different altitudes is built, and the training, validation, and evaluation of the detection models YoloV3 and YoloV4 are carried out. It is shown that YoloV4 achieved 13% more performance in real-time detections. Also, in comparisons of animal detections for each altitude, up to 40 meters away from the animals from the ground, both detectors had accuracies greater than 97%, however, after 50 meters away up to 130 meters, YoloV4 was able to maintain its accuracies above the YoloV3 results, proving to be a more effective and efficient detector for monitoring systems of endangered animals in the African savannah.

**Table 1:** Literature comparison of detectors based on convolutional neural networks for animal detection tasks. *Source* Authors.

Work	Comparative	Dataset
de Arruda et al.^26^	VGG-Net, Fast-RCNN	ImageNet
Schneider et al.^33^	Faster R-CNN, YoloV2	RTC, Snapshot-
		Serengeti
Biswas et al.^34^	DenseNet201, InceptionResNetV2	Own Dataset
	MobileNetV2, ResNet50	
	ResNet152V2, Xception	
Adami et al.^35^	YoloV3, YoloV3-Tiny	Own Dataset,
		Data augmentation
Petso et al.^30^	YoloV3, YoloV4	Own Dataset
This paper	**YoloV4**, **Scaled-YoloV4**	**BRA-Dataset**
	**YoloV5**,**YoloR**	**Data augmentation**
	**YoloX**, **YoloV7**	

In this study, we compare variations of YOLO detectors for detecting animals frequently killed on Brazilian highways. Models from other single-stage detector architectures are not included. Unlike related work, our goal is to test detection models on small datasets for supervised training. We also propose using data augmentation on the image set to increase the level of comparison between the models’ behavior in two training scenarios.

## YOLO architectures

The YOLO architecture was created in 2015 by Redmon et al.^29^, to perform detections in real-time, using the input image only once in the neural network, overcoming the challenges of two-stage object detectors created previously. This was possible due to the division of the image into grids of small sizes, which detect possible parts of a single object individually and then, with the aid of the non-maximum suppression (NMS) technique, the filter, and adjustment of the most representative bounding box on the object of interest in the image.YOLO over time has had some tweaks and in the work of Redmon, and Farhadi^31^ YoloV2 was released with the addition of some features that improved the accuracy of the network, such as, for example, Batch Normalization in all convolution layers. In addition, the resolution of the input image in the classification and detection layers was also increased, alternating the resolutions of the images in different parts of the network, allowing it to support higher resolutions than the images used for training. Anchors were also used in the convolution layers. The anchors provide pseudo-detections over a grid, having to only make comparisons at the end of the network with the anchors for the final regression of the object’s bounding box, reducing the computational cost of the first version.

**Figure 1:**
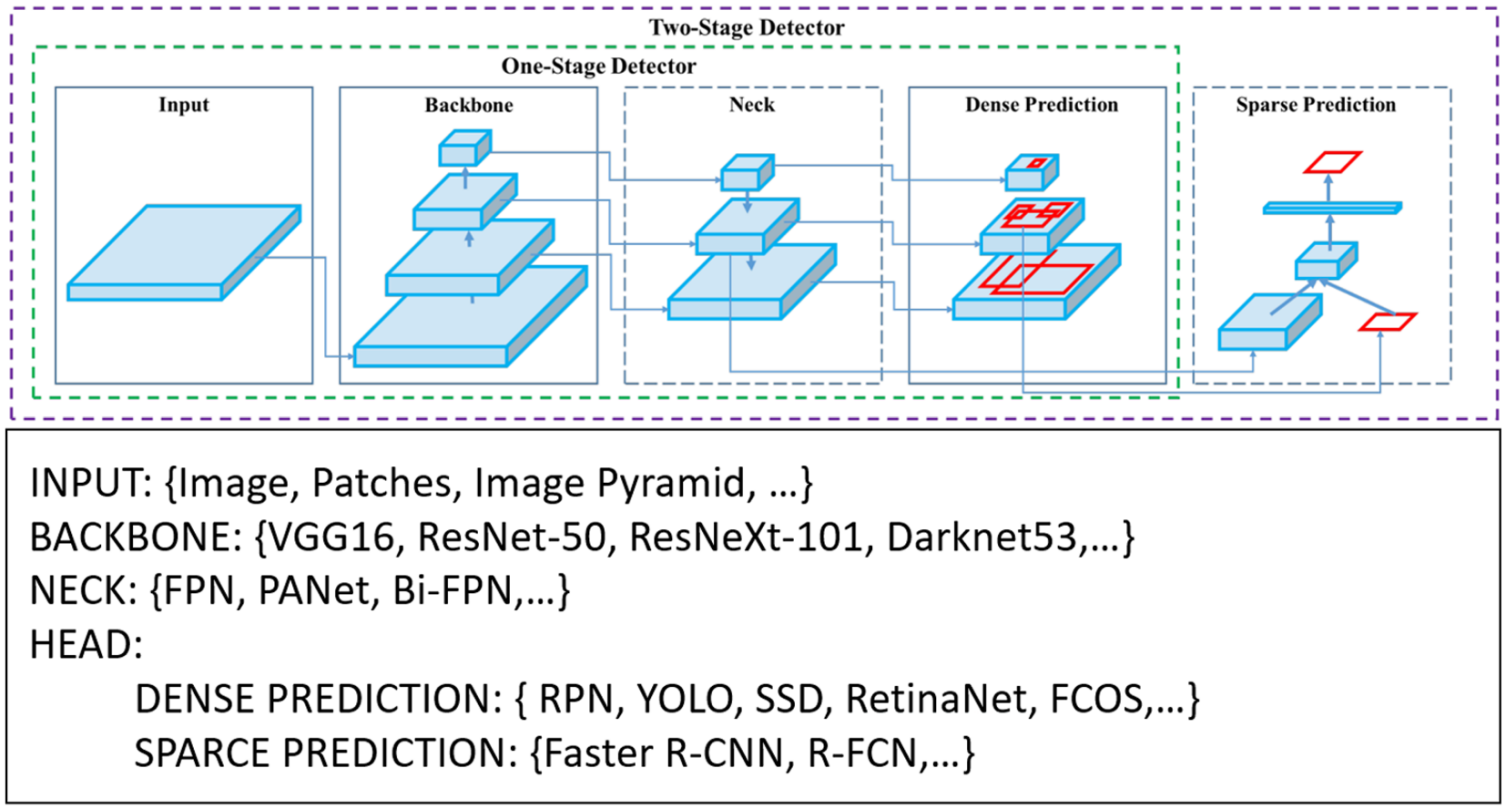
General architecture proposed by Bochkovskiy et al.^37^, focused on object detectors based on one-stage and two-stage convolutional neural networks. (Adapted image).

In the third version of Yolo (YoloV3) created by Redmon and Farhadi^36^ some of the new features are the use of independent logistic classifiers for the classes using the binary cross-entropy loss as a loss function, removing the softmax layers used in previous versions. YoloV3 also added a new internal neural network called Darknet-53, with 53 convolution layers with an increased speed due to reduced floating point operations. Another notable feature of this version is the prediction of bounding boxes at different scales, predicting three boxes for any grid, thus three different outputs of the regression. In 2020, YoloV4 was released^37^ which has up to 12% more performance than YoloV3. Other information brought by the authors of this version is the presentation of the anatomy of object detectors in general (Fig. 1), containing three parts: BackBone (convolutional neural network for feature extraction), Neck (concatenation of extracted features) and Dense Prediction (Regression step for creating bounding boxes).

Following this structure, the fourth version of Yolo presented changes in BackBone and Dense Prediction, where in addition to changing to a CSPDarknet53 network, the Bag of Freebies (BoF) was implemented, applying data augmentation techniques (mosaic images) and techniques such as smoothing and regularization of classification outputs (DropBlock regularization and Class label smoothing). The Bag of Specials (BoS) technique was also implemented, with the loss function Mish activation, even more, in the Neck layer, the use of SPP-block, SAM-block, and PAN path-aggregation block networks for the concatenation of features.

With the coming of YoloV4, it didn’t take long for YoloV5^38^ to be developed. However, YoloV5 does not have a scientific article that shows its improvement in performance compared to the previous ones. Furthermore, unlike YoloV4, the fifth version was created from the Pytorch framework made in Python and not in C, as in the Darknet framework. YoloV5 provided five types of different grid sizes (N, S, M, L, and X), for different processing power and accuracy demands and scenarios. Structurally, YoloV5 and YoloV4 are very similar in the Backbone, Neck, and Head layers. Therefore, YoloV5 became an alternative to YoloV4, but not a demonstrably better version in terms of accuracy.

With the use of PyTorch in YoloV5, there was an improvement in model training time, and this enabled an improved implementation of YoloV4, Scaled-YoloV4^39^. One of the main reasons this version is fast is the use of convolutional neural networks created following the concepts of Cross-Stage Partial Networks^40^ as in YoloV4, however, the main contribution of this version is the increase in depth and number of stages in the Backbone layers and Neck, thus improving performance in detecting large objects in high-resolution images. Another different feature of YoloV4 is that Scaled-YoloV4 uses less data augmentation on the training dataset. On the other hand, in the test set, Test Time Augmentations are made, which apply these data increases between prediction results, thus improving performance. Overall, Scaled-YoloV4 is superior in performance over YoloV4, demonstrated over MS COCO.

**Figure 2:**
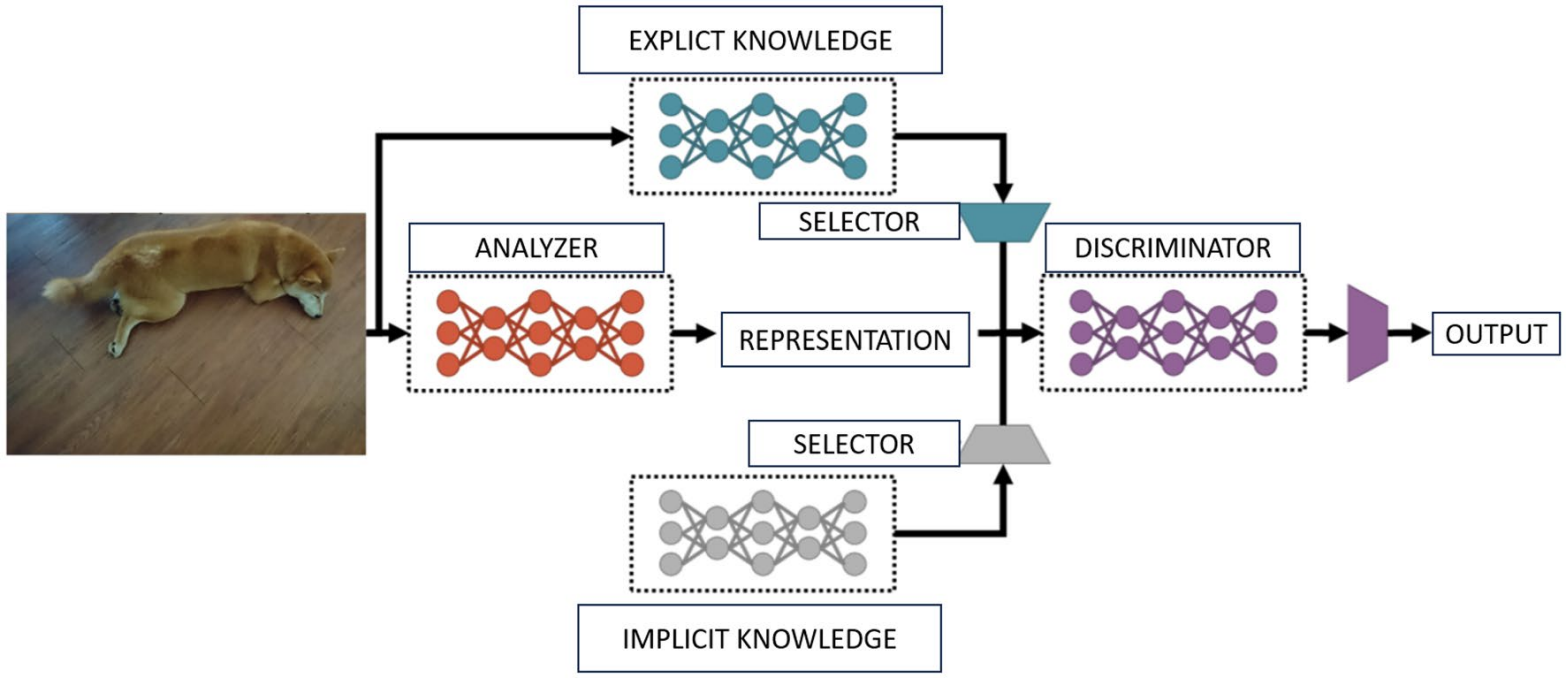
A supervised approach proposed by Wang et al.^41^ to learning (implicit learning) mixed with explicit learning, which is based on the immediate input given to the network. (Adapted image).

In the same year that the Scaled-YoloV4 version was released, some new techniques on Yolo were being implemented, such as, for example, YoloR^41^, which brings a supervised approach to learning (implicit learning) mixed with explicit learning, which is based on the immediate input given to the network. The idea of this version is to allow the machine, with a single entry, to have interpretations that serve several tasks, that is, new learning angles and not just using what was previously learned. Fundamentally, there are three parts to the idea (Fig. 2) for YoloR to work. First, the process of kernel space alignment, prediction refinement and CNN creation with multi-task learning is done. This CNN not only learns how to get the correct output but also returns the other possible coherent outputs, which represent the various interpretations of the image.

**Figure 3:**
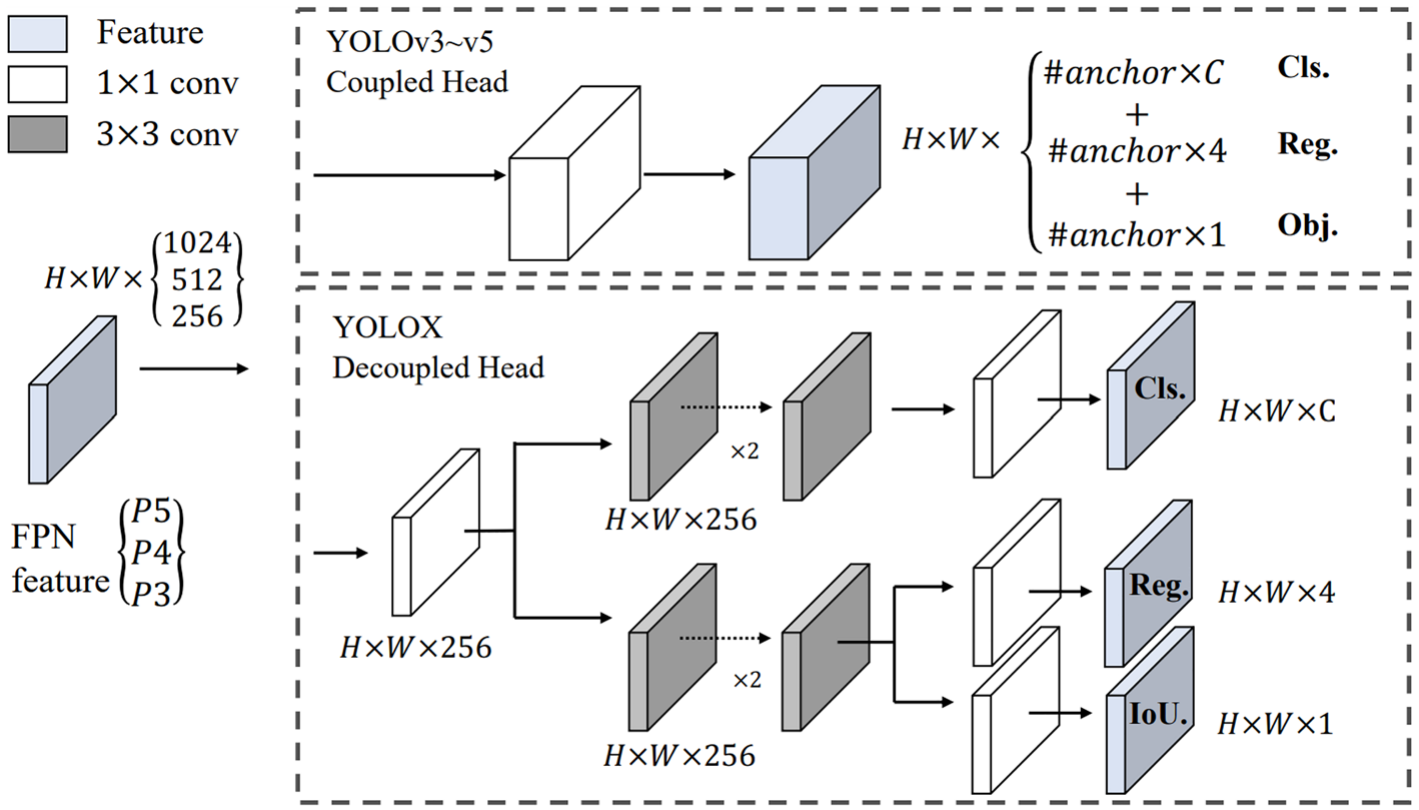
YoloX architecture proposed by Ge et al.^42^ for decoupling the classification and regression layers in the Head layer of the neural network.

Another variant is YoloX^42^ that based on YoloV3, but with some improvements, mainly for training. YoloX applies data augmentation techniques such as RandomHorizontalFlip, ColorJitter, Multi-scale, Mosaic, and MixUp on the training dataset. Another important and innovative point of this version is the non-use of anchors, which despite having been widely used by previous versions for the detection of more objects in the same grid, there are some disadvantages, such as, for example, stipulating ideal anchors before training is time-consuming with the cluster analysis method. Furthermore, the fact that detecting multiple objects on a grid can directly impact performance on certain systems. This change is illustrated in Fig. 3. Among other innovations is the implementation of SimOTA, an optimized version of the OTA (Advanced method for assigning candidate labels to objects. For more information, consult the work of Ge et al.^43^.) using the Sinkhorn-Knopp algorithm.

**Figure 4:**
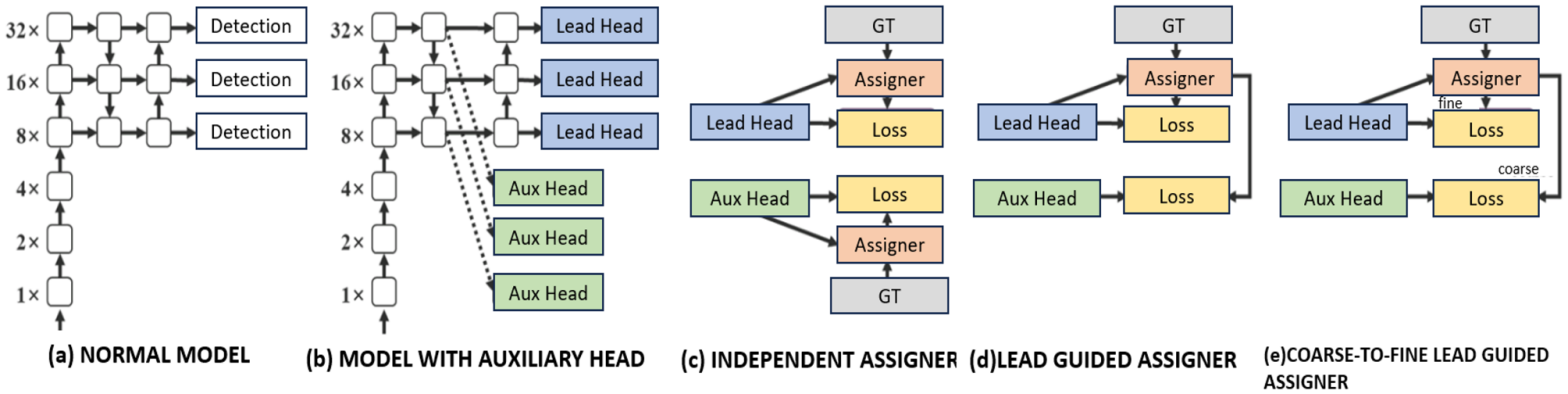
Auxiliary Head Coarse-to-Fine of architecture in YoloV7 created by Wang et al.^44^ (Adapted image).

Finally, the state-of-the-art object detection version was released in the work of Wang et al.^44^, called YoloV7, which sought to further increase the accuracy in predicting bounding boxes. In short, the main contribution of this version is the reduction of gradient propagation in the back-propagation, which correlates with the amount of memory used to store the network layers. This contribution helps speed up the learning of the network. For this, the use of an Extended Efficient Layer Aggregation Network (E-ELAN) was proposed in its architecture. Another contribution is that YoloV7 scales its models in depth, width, and resolution while concatenating the layer outputs. Re-parameterization is also used in YoloV7, it allows weights to become more robust in identifying the general characteristics of the model to be created. Furthermore, YoloV7 implements Auxiliary Head Coarse-to-Fine in the middle band of the network. They are auxiliary Head layers to supervise the course of future detections that will be performed in the final layers. They are not as accurate as future object predictions, but they do indicate how the model might be behaving during training. This strategy can be seen in Fig. 4.

Overall, YoloV7 has proven its speed and accuracy among all known real-time detectors that perform within 5 FPS to 160 FPS. In addition to the contributions highlighted above, its accuracy reached a result of 56.8% AP on the MS COCO Dataset validation set, becoming a YOLO strategy benchmark, having superior results when compared to architectures such as YOLOR, YOLOX, Scaled-YOLOv4, YOLOv5, DETR, Deformable DETR, DINO-5scale-R50 and others in detection speed tests on videos with different scales and resolutions.

## Methodology

The methodology used in this work follows 3 fundamental steps for the comparison of detection technologies based on YOLO.

**Figure 5:**
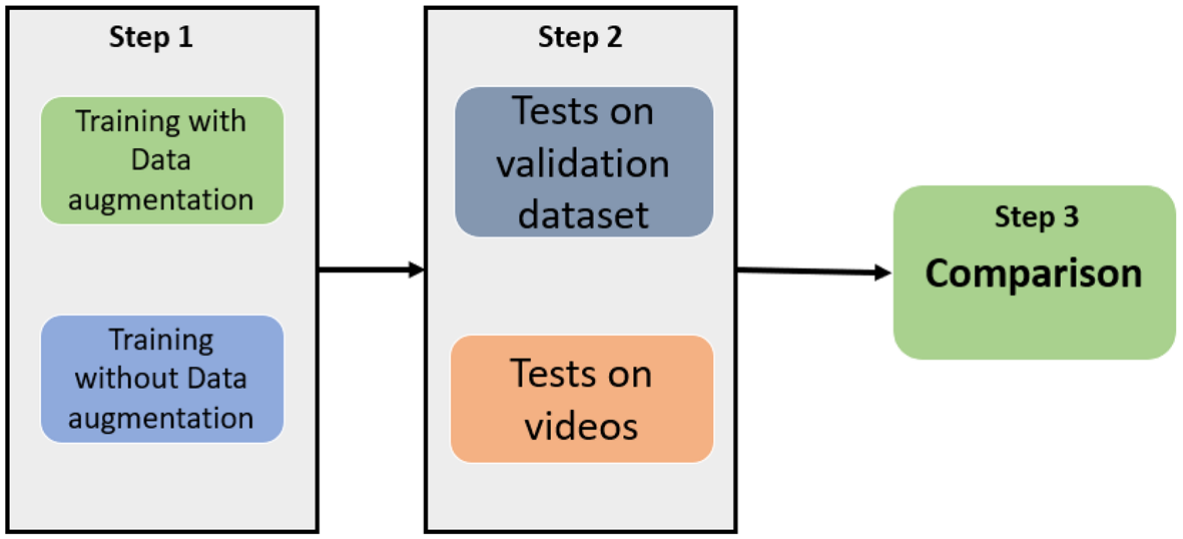
Proposed method for evaluating target object detector architectures. The method consists of carrying out training, testing, and comparison using performance metrics.

Figure 5 presents the steps for carrying out comparisons between the different YOLO models. In Step 1, two types of training are configured and carried out. Each model was first trained on 80% of the BRA-Dataset (a total of 1458 images without data augmentation) and then trained again on the same set with data augmentation, resulting in 8407 images after augmentation (Step 1). The augmentation techniques applied to the training set were: Horizontal Shift, Vertical Shift, Horizontal Flip, Vertical Flip, and Rotation. Table 2 presents the application configuration of each technique and its hyperparameters. The choice of parameters was arbitrary and experimental.

**Table 2:** Setup of hyperparameters for each data augmentation applied in the BRA-Dataset.

Augmentation technique	Hyperparameters
Horizontal shift	Shift = 0.7 (ratio multiplier) * width (in pixels)
Vertical shift	Shift = 0.6 (ratio multiplier) * height (in pixels)
Horizontal flip	Flips all rows and columns horizontally along the y-axis
Vertical flip	Flips all rows and columns vertically along the x-axis
Rotation	45° rotation angle

The choice of parameters was arbitrary and experimental. In the hyperparameters column, it is shown which transformation is performed on the image, according to the arbitrary variables together with the image properties (dimensions and pixel scanning axes).

The chosen data augmentation acts as a form of regularization (a technique to avoid overfitting). With data augmentation, the model has access to a greater amount of varied information, which can prevent it from focusing too much on specific features present in a limited set of training images. Furthermore, the application of data augmentation aims to mitigate the problem of initial overfitting (Early overfitting is a phenomenon where a machine learning model overfits training data early in the training process, before converging on a general, valid representation of the problem. This can happen for a number of reasons and can be especially problematic in scenarios where the training set size is small or the data characteristics are complex.), which is directly related to excessive variance. In which a model is able to quickly and overfit the training data, including the noise in the data. This means that at the beginning of training, the model may perform excellently on the training data, but perform poorly on new data (test or validation set), indicating a lack of generalization.

Both pieces of training have as input images 416x416 dimensions and 100 epochs per model. The training was based on the use of transfer learning, with pre-trained models provided by the authors of each architecture, which vary between pre-trained models in the MS COCO dataset. Moreover, for all models, except for the YoloV4 and YoloR model, in addition to the conventional version of the architecture, its versions with smaller networks and versions with large and complex networks are trained (e.g, YoloV5-N, YoloV5-S, YoloV5-M, YoloX-M, YoloV5-L, YoloX-L, Scaled-YoloV4-P5, YoloV5-X, YoloX-X, Scaled-YoloV4-P6 and YoloV7-X). The models with nomenclature containing “N”, “S”, “M”, “L” and “X”, refer to the depth and complexity of the network, such as “Nano”, “Small”, “Medium”, “Large” and “eXtreme”, respectively. For Scaled-YoloV4, the “p5, p6” at the end of the model name, indicates the number of image scaling layers. But, for YoloR, the default version contains “‘p6” in the nomenclature, but does not refer to the depth. All training was performed using dedicated Nvidia RTX 3060 GPU, with 12GB of exclusive memory and assistance from the CUDA module. For the light versions used, the training time was 2 h with the database without augmentation, for the database with augmentation, the training time was 4 h.

Step 2 consists of carrying out the tests with the trained models. First, tests are performed on the BRA-Dataset validation set, which contains 363 images, with 403 labels. It is proposed to test the inference speed of the models using GPU and edge device on videos of animals (To access the videos, contact the authors.), recorded in the ecological park of São Carlos, Brazil and free videos on the internet (for classes that were not included in the ecological park). The BRA-Dataset and videos were chosen as the validation set because they contain instances of animals in occluded environments, such as vegetation and cages, and in poses that do not favor detection. Additionally, some animals are not fully visible in the images.

In Step 3, the results of the proposed tests are compared by summarizing the precision, recall, and mAP metrics in BRA-Dataset achieved by the models. Additionally, a qualitative analysis is conducted on the models’ performance on selected videos in terms of detecting animals in situations of occlusion, small and distant objects, and in videos with poor image quality. This allows for insights beyond the quantitative metrics typically analyzed in the literature.

### BRA-dataset

**Figure 6:**
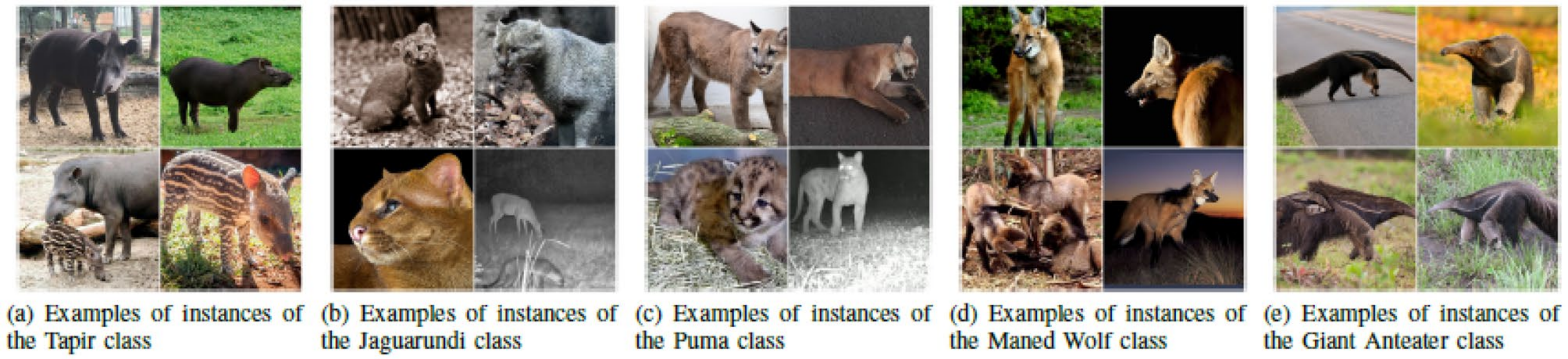
All classes BRA-Dataset^32^ supports. The classes are medium and large animals with a high risk of extinction in the Brazilian fauna.

The Brazilian Road’s Animals Dataset (BRA-Dataset)^32^) is an open and free dataset exclusively featuring animal species from the Brazilian fauna that are commonly hit on highways. The dataset contains 1823 images in all and about five classes of medium/large animals. The tapir, the Jaguarundi, the Maned Wolf, the Puma and the Giant Anteater, the species can be seen in Fig. 6.

The species in the BRA-Dataset are found in biomes such as the Brazilian Cerrado, the Pantanal, and the Atlantic Forest. In short, these animals often appear on the sides of roads to cross and are run over due to several factors. One of the crucial factors for accidents with animals to occur is that vehicles have headlights that can distort the animal’s vision and reflection, another factor is the sound noise generated by different vehicles in two directions, which confuses the animal during the crossing. The BRA-Dataset has images in JPG formats and is labeled in YOLO Darknet and Pascal VOC formats, widely used in labeling computer vision datasets.

The BRA-Dataset was built by applying a methodology that allows the construction of datasets quickly and cheaply since it uses the Google Images search engine to filter images from free internet sources for its design. Although the method of its construction proposed a data cleaning step, there was no removal of images of real animals that could possibly negatively affect the training of detection models, such as animals in unfavorable poses (animals in back view, top view, or zoomed) and images with low quality, because the BRA-Dataset cleaning criterion consisted of removing images of non-real animals and images with noise. In addition, the dataset does not have images of animals in scenarios of complete or partial occlusion, nor in unfavorable weather conditions, due to BRA-Dataset using images that must contain the animal clearly. Therefore, due to the methodology proposed for its construction, the variety of quality, dimension of the images and poses, and zoom of the animals are high, but in general, for most images of all classes, the animals are presented in the side view, and the other images, have zoom applied to the face and trunk.

## Results

This section presents the results of the animal detector tests in the proposed scenarios, i.e., evaluation on the validation dataset and on video recordings. First, we present the results of the models using the same test metrics for their respective training. The results for the BRA-Dataset validation set are then presented. In all the result tables, some entries are marked with an asterisk (*), indicating a potential overfitting. This risk can occur when training supervised models with limited data due to a lack of variation in characteristics. This issue was identified by observing exceptional and unexpected results, such as metrics reaching values close to 100% or precision and recall values that are vastly different (this can be better observed in the class results table, Table 4).

### Evaluation on the validation dataset

For the evaluation, models are run with a confidence threshold and an IoU threshold of 50%. Table 3 presents the results of the models without and with data augmentation on the validation set. When considering the models without data augmentation, in the vast majority, the models were negatively affected by the little data provided for training. For overfitting validation, the criterion used to determine whether models without data augmentation were potential models with overfitting refers to the comparison of their results with the results achieved after data augmentation in the BRA-Dataset. It was used as a criterion if the models presented all three observed metrics with values above the baseline achieved after data augmentation in the same model, defining that there was overfitting. If at least one of the metrics has a result equal to or less than that achieved after data augmentation is applied, the model in question would not be categorized as potentially overfitting. It was expected that the application of data augmentation would not allow a reduction in performance in the metrics, if this occurs, it would be in scenarios in which the model trained without augmentation was experiencing early overfitting. As an exception, the tested weights of the YoloV7 architecture did not follow this rule, due to its sensitive characteristic to the increase of simple data, which is not similar to other architectures. Therefore, the conventional YoloV7 model was categorized as potentially overfitting, given its low generalization to videos without any challenge (explained in more detail in the Evaluation on videos subsession).

Theremore, the Table 4 shows the specific results for the five classes of the BRA-Dataset in the trained models without data augmentation. The results support the categorization of overfitting in the models (this condition was also confirmed by analyzing the models’ loss curves). Some models achieved 100% precision or recall, confirming our expectations.

**Table 3:** Overall Precision, Recall, and mAP@50 results for the detection models trained without and with data augmentation.

Model	Without data augmentation			With data augmentation		
	Precision	Recall	mAP@50	Precision	Recall	mAP@50
YoloV4*	0.96	0.96	97.4	0.89	0.75	**89.4**
Scaled-YoloV4-p5*	0.98	0.96	95.9	** 0.94**	0.83	81.4
Scaled-YoloV4-p6*	0.97	0.96	96.2	0.91	**0.84**	82.5
YoloV5-N*	0.97	0.93	96.9	0.88	0.71	80.9
YoloV5-S*	0.98	0.93	96.9	0.89	0.72	82.4
YoloV5-M*	0.97	0.93	96.7	0.88	0.74	83.4
YoloV5-L	0.94	0.91	83.5	0.91	0.75	84.8
YoloV5-X	0.95	0.90	84.3	0.91	0.80	87.0
YoloR-p6*	0.93	0.96	98	0.90	0.83	88.8
YoloX-M	0.65	0.72	65.8	0.65	0.71	65.3
YoloX-L	0.66	0.73	66.8	0.66	0.73	66.5
YoloX-X	0.67	0.72	67.0	0.67	0.72	67.7
YoloV7*	0.72	0.58	65.8	0.83	0.60	56.7
YoloV7-X	0.79	0.70	77.4	0.80	0.65	61.8

Models with an asterisk mark (*) indicate a potential overfitting. The highest values without data augmentation are not highlighted in bold, as they could mistakenly lead the reader to believe these results are positive or desirable. Therefore, not highlighting them can help avoid this problem. The result of models with data augmentation, in bold, the best results for each metric observed.

**Table 4:** Class-specific results of Precision, Recall and mAP@50 respectively for detection models trained without data augmentation.

Model	Giant-anteater	Jagua rundi	Maned-wolf	Puma	Tapir
YoloV4*	0.97 | 0.94 | 96.8	0.93 | 0.97 | 96.8	0.96 | 0.91 | 95.8	0.98 | 0.96 | 98.8	0.97 | 0.99 | 98.8
Scaled-YoloV4-p5*	1.00 | 0.92 | 92.2	0.97 | 0.98 | 98.4	0.97 | 0.93 | 93.4	1.00 | 0.95 | 95.5	0.98 | 1.00 | 99.5
Scaled-YoloV4-p6*	1.00 | 0.94 | 94.5	0.97 | 0.97 | 97.5	0.96 | 0.96 | 96.2	0.97 | 0.94 | 94.5	0.97 | 0.98 | 98.5
YoloV5-N*	0.99 | 0.91 | 95.6	0.95 | 0.94 | 96.2	0.97 | 0.93 | 96.1	0.97 | 0.90 | 97.1	0.96 | 0.98 | 99.4
YoloV5-S*	0.98 | 0.91 | 94.7	0.97 | 0.94 | 97.4	0.97 | 0.93 | 96.0	0.98 | 0.91 | 96.8	0.98 | 0.97 | 99.4
YoloV5-M*	0.99 | 0.92 | 95.9	0.97 | 0.92 | 97.0	0.97 | 0.92 | 95.7	0.95 | 0.92 | 95.7	0.98 | 0.97 | 99.4
YoloV5-L	0.90 | 0.82 | 85.8	0.87 | 0.82 | 88.5	0.81 | 0.78 | 82.0	0.72 | 0.73 | 78.7	0.86 | 0.70 | 82.6
YoloV5-X	0.94 | 0.79 | 89.2	0.87 | 0.86 | 91.0	0.88 | 0.75 | 83.3	0.76 | 0.74 | 77.2	0.86 | 0.76 | 80.9
YoloR-p6*	0.95 | 0.94 | 97.4	0.93 | 0.97 | 98.4	0.94 | 0.95 | 96.3	0.89 | 0.96 | 98.3	0.96 | 1.00 | 99.5
YoloX-M	0.69 | 0.74 | 69.6	0.69 | 0.76 | 69.7	0.64 | 0.70 | 64.0	0.61 | 0.71 | 61.6	0.64 | 0.70 | 64.0
YoloX-L	0.72 | 0.77 | 72.4	0.69 | 0.77 | 69.9	0.66 | 0.72 | 66.4	0.59 | 0.69 | 59.7	0.65 | 0.69 | 65.2
YoloX-X	0.71 | 0.76 | 71.5	0.69 | 0.73 | 69.8	0.66 | 0.71 | 66.2	0.64 | 0.71 | 64.1	0.66 | 0.70 | 66.7
YoloV7*	0.75 | 0.58 | 68.7	0.78 | 0.72 | 78.0	0.79 | 0.65 | 70.1	0.59 | 0.47 | 51.3	0.70 | 0.50 | 60.9
YoloV7-X	0.81 | 0.70 | 80.9	0.85 | 0.77 | 82.7	0.79 | 0.76 | 78.7	0.59 | 0.66 | 66.5	0.93 | 0.59 | 78.1

Models with an asterisk mark (*) indicate a potential overfitting. High values were not highlighted in bold, as they could lead the reader to mistakenly believe that these results are positive or desirable, therefore, we believe that not highlighting them can help avoid this problem.

Table 5 shows the class-specific results obtained from the detection models trained with augmented data on the validation set. The results are more consistent with the reality of machine learning models and do not reach exorbitant values. For the Tapir class, some models achieved 100% precision. To verify that there was no overfitting during training, the loss curves of the YoloV4-Scaled-p5, YoloV5-L and YoloR-p6 models were analyzed and no overfitting was observed. When the BRA-Dataset validation is observed in more detail, it is possible to observe that for the Tapir class, there are approximately ±40% of the images with the animal on its side, which resemble the training images, causing high precision. On the other hand, the Puma class generally had the lowest results in both tests with the validation set. Upon closer examination of the Puma class training set, it was observed that there is an abundance of images with sitting or lying animals or images showing only the animal’s face. This directly impacts the collection of class characteristics and impairs classification.

In general, in animal detection systems on highways, it is important to know the recall level of the models. This metric emphasizes the problem of having many false negatives, meaning animals that are present on the highway but not detected or classified by the model. Low recall may result from few detections in positive situations and can directly impact, for example, animal counting and population estimation for a specific road or region. It can also result in a lack of alertness for an animal on a certain stretch of road, leading to a failure to register the presence of the animal and potentially hindering rescue efforts.

**Table 5:** Specific results by class of Precision, Recall and mAP@50 respectively for detection models trained with data augmentation.

Model	Giant-anteater	Jagua rundi	Maned-wolf	Puma	Tapir
YoloV4	0.93 | 0.76 | 90.8	0.93 | 0.76 | 92.7	0.94 | 0.77 | 88.6	0.82 | 0.70 | 84.5	0.88 | 0.77 | 90.6
Scaled-YoloV4-p5	0.92 | 0.85 | 81.9	0.92 | 0.89 | 88.9	0.97 | **0.81** | 80.5	0.89 | 0.79 | 76.3	1.00 | 0.79 | 79.5
Scaled-YoloV4-p6	0.91 | 0.83 | 80.0	0.96 | 0.88 | 87.6	0.87 | **0.81** | 79.4	0.84 | **0.84** | 82.2	0.97 | **0.83** | 83.4
YoloV5-N	0.87 | 0.70 | 80.0	0.88 | 0.80 | 86.5	0.93 | 0.68 | 81.4	0.77 | 0.62 | 71.3	0.87 | 0.70 | 80.0
YoloV5-S	0.92 | 0.69 | 80.3	0.87 | 0.83 | 87.6	0.85 | 0.72 | 82.3	0.84 | 0.70 | 78.1	0.95 | 0.69 | 83.4
YoloV5-M	0.91 | 0.76 | 84.6	0.86 | 0.77 | 85.5	0.90 | 0.76 | 85.5	0.77 | 0.71 | 76.7	0.95 | 0.71 | 84.7
YoloV5-L	0.93 | 0.77 | 85.5	0.92 | 0.89 | 93.4	0.85 | 0.72 | 82.2	0.87 | 0.69 | 79.5	1.00 | 0.66 | 83.3
YoloV5-X	0.86 | 0.77 | 83.7	0.95 | **0.92** | 95.3	0.91 | 0.79 | 86.4	0.89 | 0.83 | 86.0	0.95 | 0.69 | 83.5
YoloR-p6	0.88 | **0.86** | 89.2	0.89 | 0.88 | 92.4	0.92 | 0.78 | 87.4	0.84 | 0.80 | 83.8	1.00 | 0.82 | 91.1
YoloX-M	0.66 | 0.73 | 66.7	0.67 | 0.72 | 67.0	0.64 | 0.69 | 64.5	0.60 | 0.68 | 60.2	0.67 | 0.72 | 67.8
YoloX-L	0.69 | 0.78 | 69.8	0.67 | 0.73 | 67.6	0.66 | 0.72 | 66.6	0.63 | 0.71 | 63.4	0.64 | 0.70 | 64.7
YoloX-X	0.70 | 0.76 | 70.5	0.70 | 0.74 | 70.8	0.68 | 0.73 | 68.6	0.63 | 0.71 | 63.3	0.65 | 0.68 | 65.0
YoloV7	0.95 | 0.63 | 61.9	0.88 | 0.70 | 67.5	0.82 | 0.57 | 56.1	0.78 | 0.56 | 47.7	0.72 | 0.56 | 50.1
YoloV7-X	0.78 | 0.66 | 62.8	0.85 | 0.77 | 74.8	0.89 | 0.62 | 61.4	0.74 | 0.59 | 54.0	0.76 | 0.60 | 55.8

The results of highest recall per class evaluated are highlighted in bold, indicating which model performed best in reducing false negatives per class.

Considering only the results after applying data augmentation, and exclusively observing the recall metric, the model that obtained the best overall performance against false negatives is Scaled-YoloV4-p5 and Scaled-YoloV4-p6 and the worst is the recent YoloV7. The fact that Scaled-YoloV4 obtained the best results in general can be attributed to the ability to manipulate different scales of the same image, allowing to obtain better characteristics, however, this ability reflects the low execution speed compared to the other versions. As for YoloV7, it is observed that due to its ability to apply native data augmentation using combination and aggregation techniques, it provides, in scenarios with other more basic techniques, a representation of characteristics of superimposed objects, that is, the architecture is sensitive to the use of data augmentations that are not part of the BoF.

**Figure 7:**
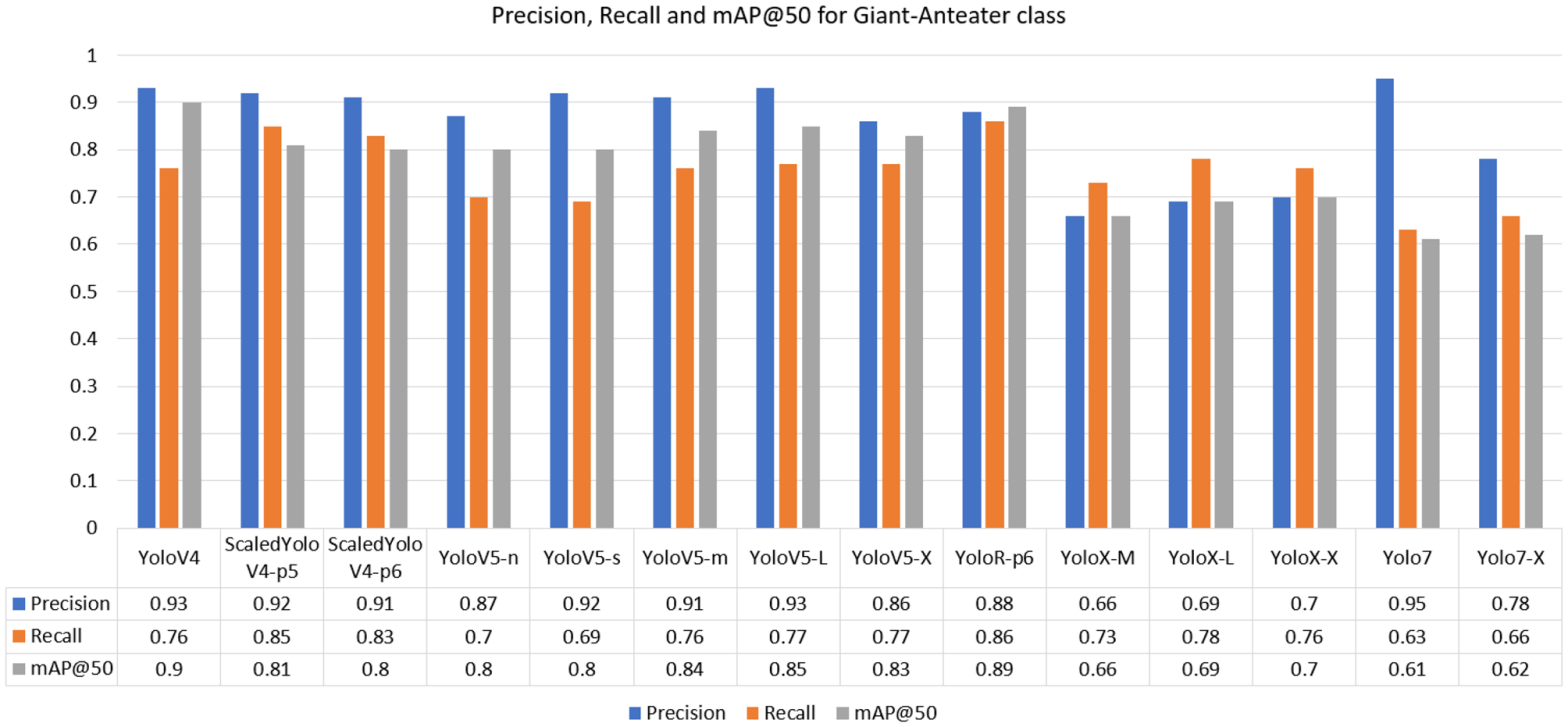
Precision, Recall and mAP@50 for the Giant Anteater class from tests with models trained with data augmentation.

**Figure 8:**
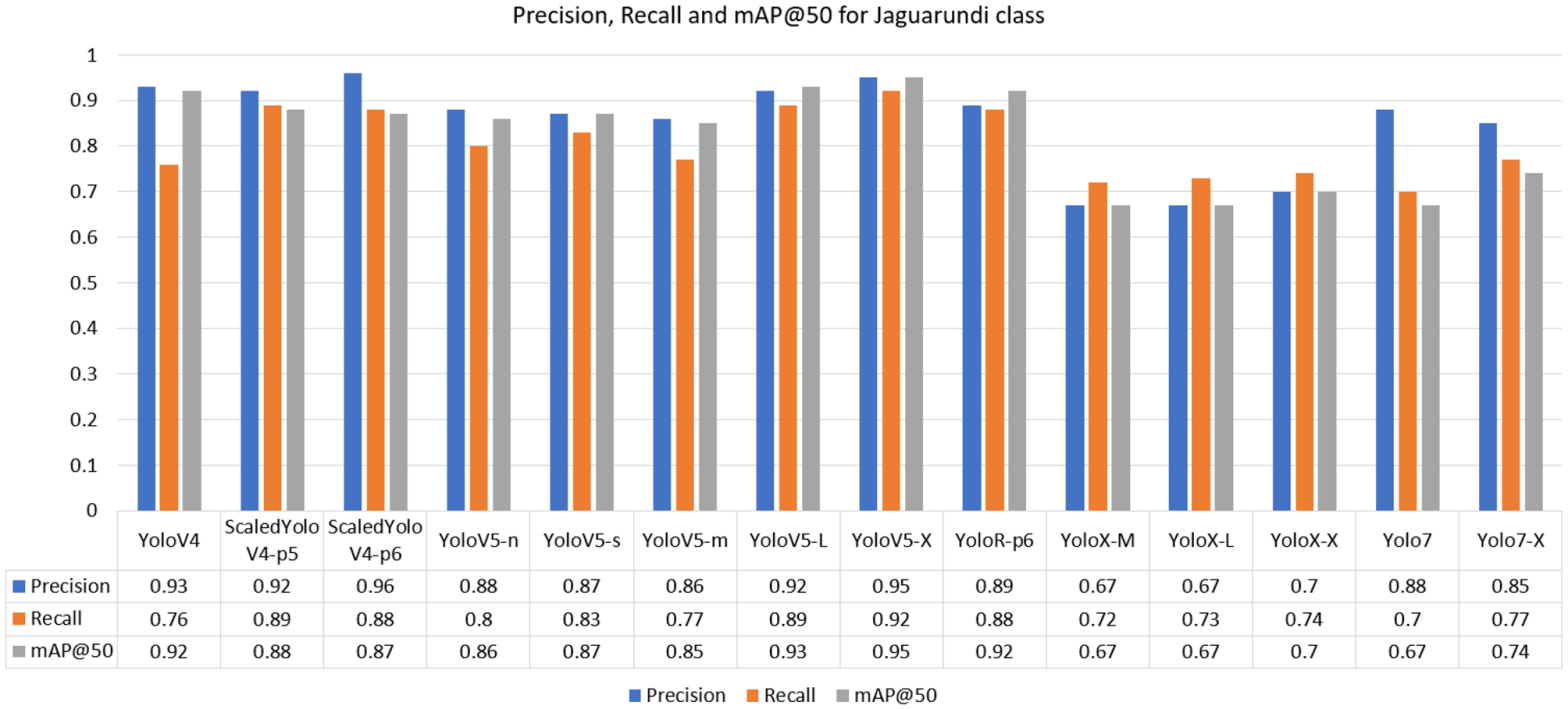
Precision, Recall, and mAP@50 for Jaguarundi class from tests with models trained with data augmentation.

**Figure 9:**
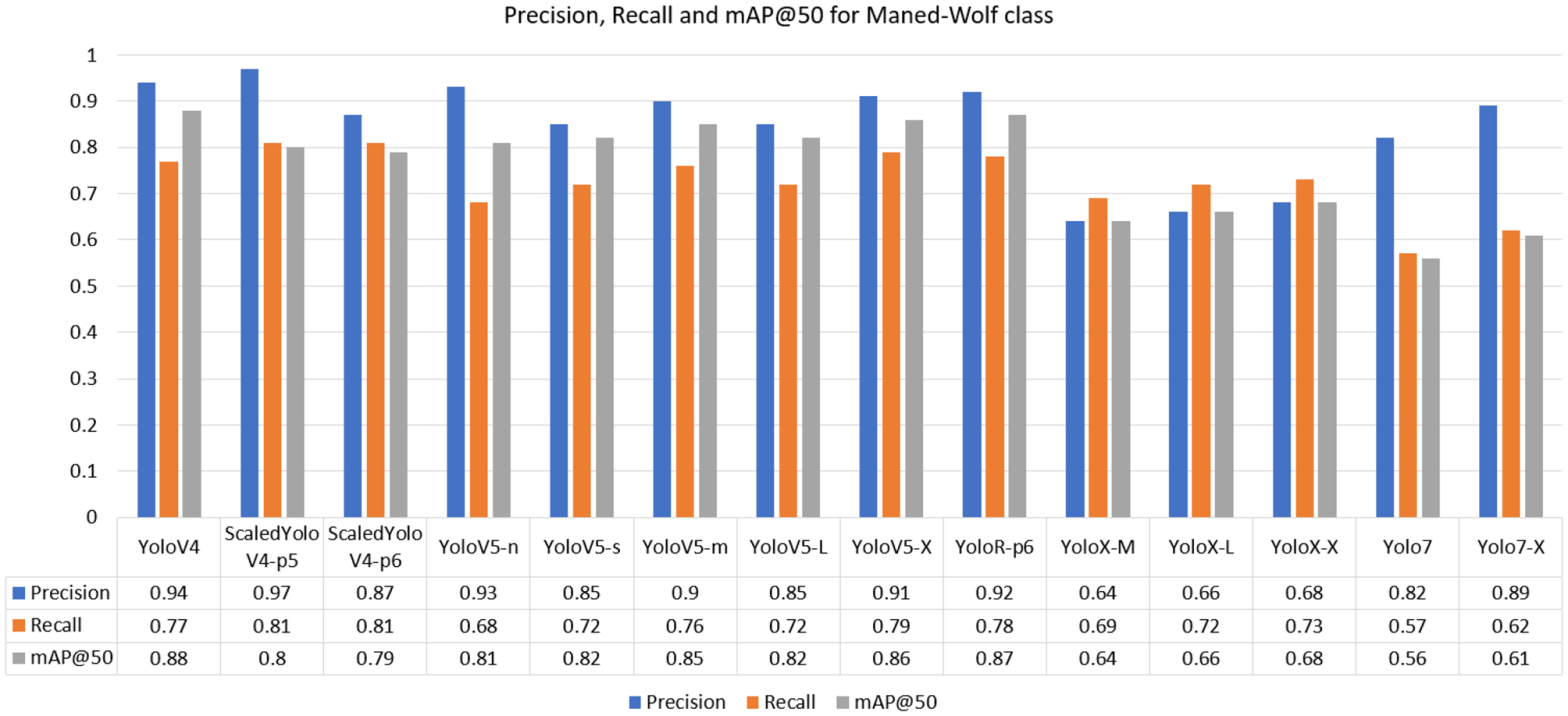
Precision, Recall, and mAP@50 for Maned-Wolf class from tests with models trained with data augmentation.

**Figure 10:**
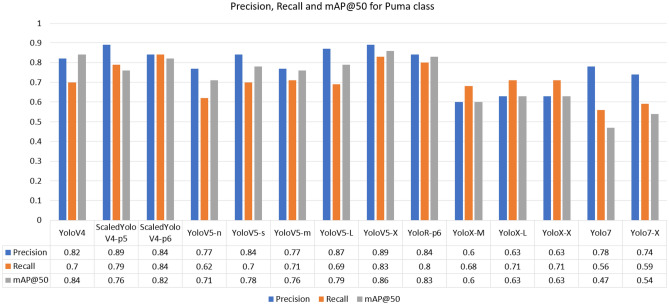
Precision, Recall, and mAP@50 for Puma class from tests with models trained with data augmentation.

**Figure 11:**
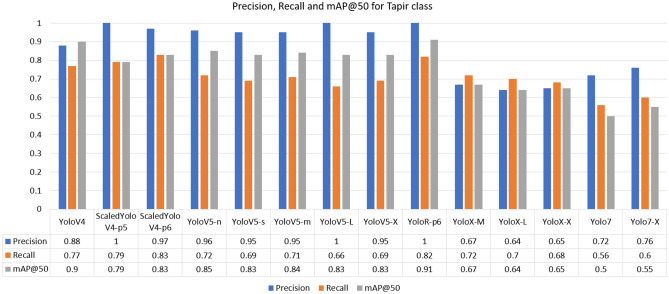
Precision, Recall, and mAP@50 for Tapir class from tests with models trained with data augmentation.

For the Giant-Anteater class, the highest recall was achieved by the YoloR architecture. The fact that YoloR obtained better performance for the Giant Anteater class reflects the use of knowledge unification layers, where there may be animals of the same class with different characteristics (black and brown Giant Anteater, young and adult Maned-Wolf, Jaguarundi with three color variations). The single representation strategy is a good alternative in these cases. For the Jaguarundi class, the holder of the highest recall was obtained at YoloV5-X weight. In the case of the Jaguarundi class, the heaviest version of YoloV5 (“x”) provided a result slightly above the Scaled-YoloV4 and YoloR architectures, which had already presented good median results for other classes. This is due to the characteristic of YoloV5 dealing well with large objects, since looking at the BRA-Dataset as a whole, many images of this class present the animal in most of the image. In the Maned-Wolf, Puma, and Tapir classes, the YoloV4-Scaled versions achieved the highest recall performances. In terms of accuracy, the best overall performance was obtained by the YoloV4-Scaled-p5 model. For the mAP metric, the classic YoloV4 model took first place, followed by the YoloR model. Below in Figs. 7, 8, 9, 10 and 11 show the performance bar graphs of each architecture for each class.

Analyzing the classes, the architecture with the lowest performance was YoloV7, reaching a value of 0.56 for recall in the Puma class. Otherwise, the best recall was obtained for the Jaguarundi class with the YoloV5-X model. In all classes, the lowest precisions were obtained by the YoloX architecture, unlike the scaled architectures of YoloV4 and YoloR, which obtained high values.

### Evaluation on videos

**Figure 12:**
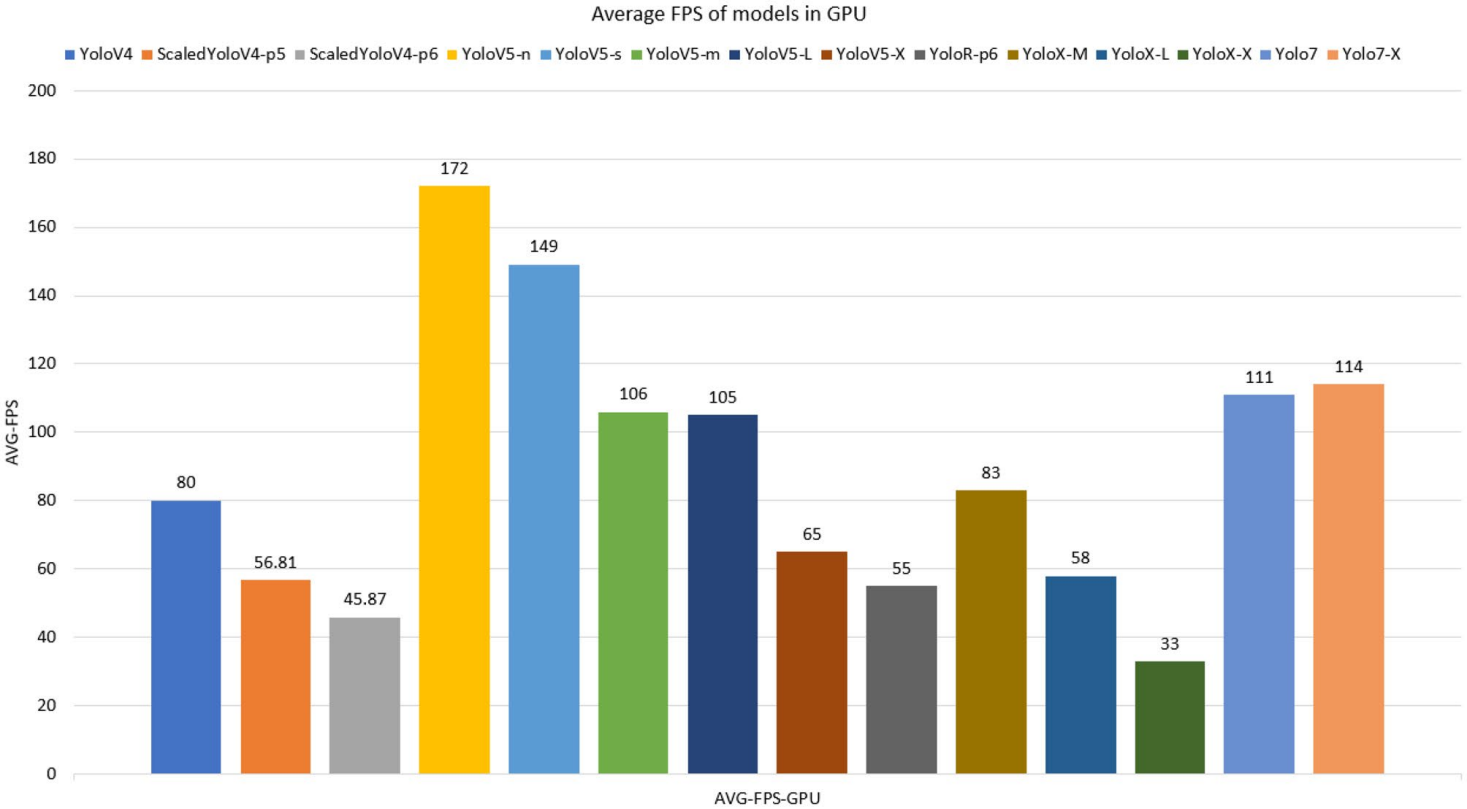
Average FPS of models trained with data augmentation run via dedicated GPU.

**Figure 13:**
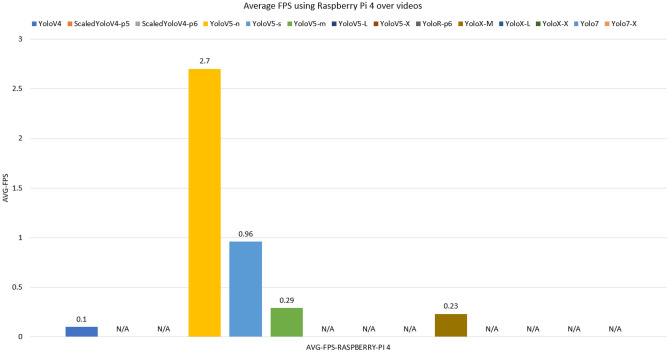
Average FPS of data augmentation-trained models run via Raspberry Pi 4 edge computing device with 1GB of RAM.

Tests with videos on the 12GB Nvidia RTX 3060 GPU were evaluated on the average FPS for each of the models. In addition, models on the Rasberry Pi 4 edge computing device with 1GB of RAM are also tested. Figures 12 and 13 shows the performance of the models on inference tests. In general, models with simpler and shallower networks are faster than those with more complex and deeper networks.

**Figure 14:**
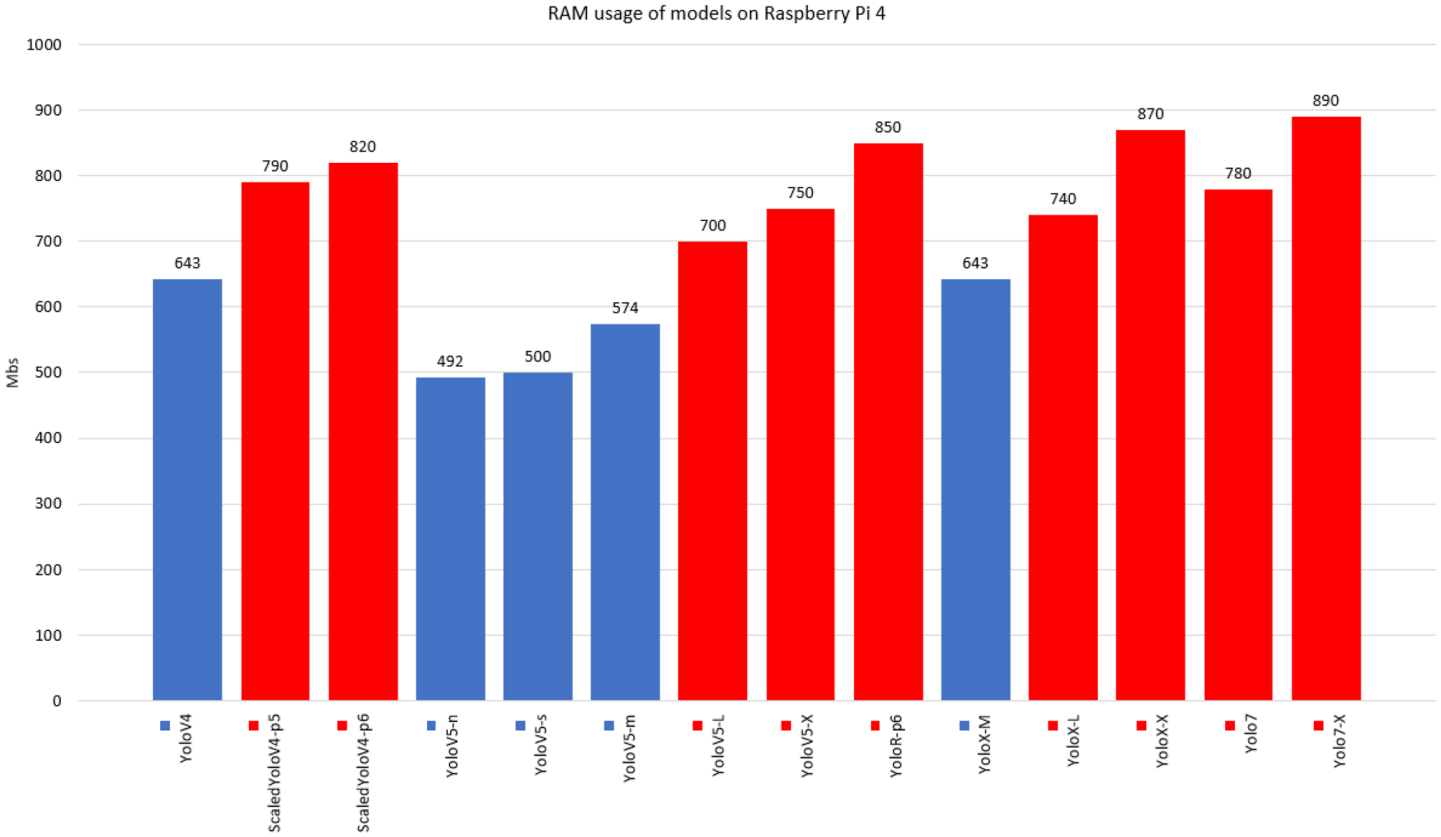
RAM usage of models on Raspberry Pi 4. Values in color red mean that the model exceeded 700Mb of RAM consumption and consequently it was not possible to execute on the device.

**Figure 15:**
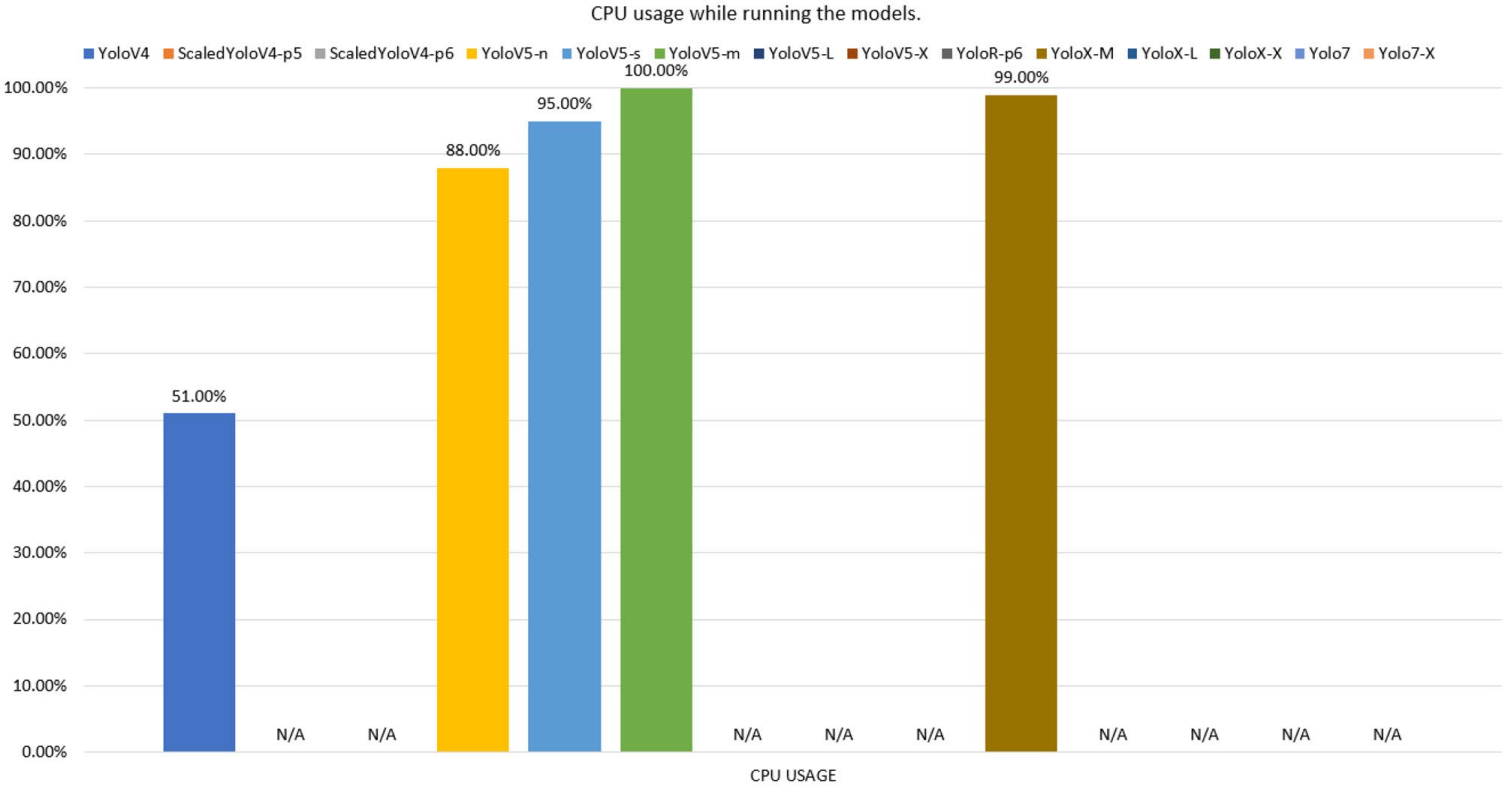
CPU usage while running the models.

In contrast, accuracy is higher in complex models. For Raspberry, the models without results demonstrate that it was not possible to load the respective weights, where it can be seen that the models that exceeded 700mb of consumption (red bars) did not allow to be executed, Figure 14 displays the memory consumption for each model. This problem occurred due to processes related to the operating system and internal processes of the mini-computer, which consumed approximately 30% of memory space. The input videos were resized from full hd to dimensions of 360 $\times$ 680 so that there was less memory usage, however most models were not loaded, requiring memorys with more space. The models that allowed the execution (YoloV4, YoloV5-n, YoloV5-s, YoloV5-m and YoloX-m) were not achieved satisfactory results, where the best FPS result was for YoloV5-n, with 2.5 FPS, with little fluidity. The CPU consumption of the models can be seen in Fig. 15. Despite the Raspberry’s high CPU usage during execution, it was not enough to read frames quickly, due to the low clock rate and few cores in its hardware composition.

For the results on GPU, the fastest model is the nano version of YoloV5, this version is the lightest and is a great alternative for mobile devices and limited edge computing. The YoloV5-N and YoloV5-S models were the only smaller models trained in order to present the difference between medium and wide models, being used as a performance ceiling. Heavier models, on the other hand, required more processing and did not achieve great performances, but they are possible to be used, the YoloV7 and YoloV7X models stand out, which, despite being complex, reached higher FPS than the other medium and wide networks. The YoloV4 architecture achieved reasonable performance even compared to later released architectures. In general, even with the lowest performing YOLO (YoloX-X), it was still possible to obtain an acceptable execution speed for inference.

For general qualitative analysis, the videos present classic computer vision challenges, such as animal occlusion (as seen in a video with the Tapir class, Fig. 17), animals far from the capture camera, and animals that camouflage themselves in their environment during video recording (as seen in a video with the Giant Anteater class, Fig. 18). None of the models trained in this study performed well on these challenges. Additionally, it was also observed that the animal’s pose also influenced detection during occlusion. The models performed well when the animal was on its side during occlusion, but struggled when the animal was on its back and occluded, resulting in increased classification errors for the class. An important observation is that in videos with animals without any obvious challenge, the detectors were able to detect and classify each class. However, the conventional YoloV7 weight (trained without data augmentation) was not successful even on these clean videos, so it was categorized as overfitting problem (Table 3).

**Figure 16:**
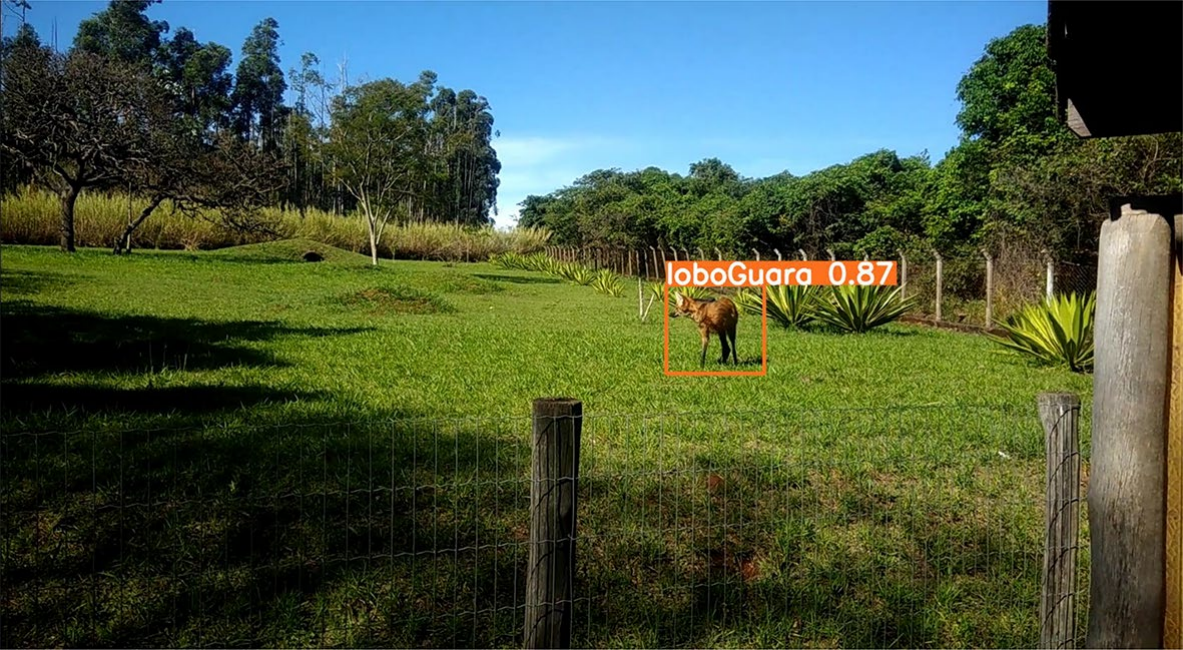
Example of a non-challenging video for the Maned Wolf class, in which the animal is visible and in a favorable position for capturing characteristics.

**Figure 17:**
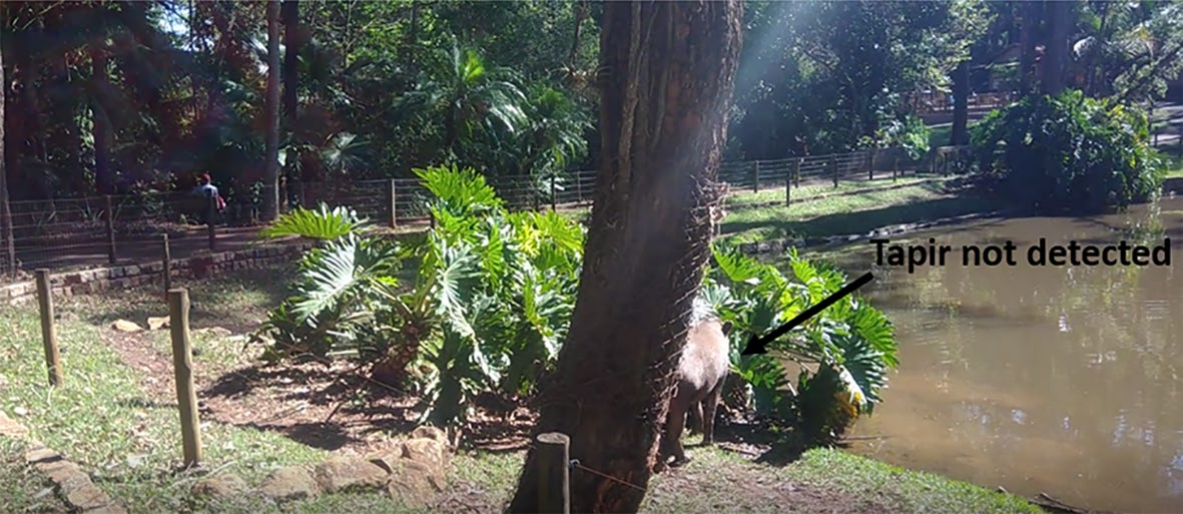
An example of a situation with an occlusion challenge on an animal from the Tapir class. The tree prevents the complete capture of the animal in the image. Furthermore, the animal has a similar color to the tree, making it even more difficult for models to interpret.

**Figure 18:**
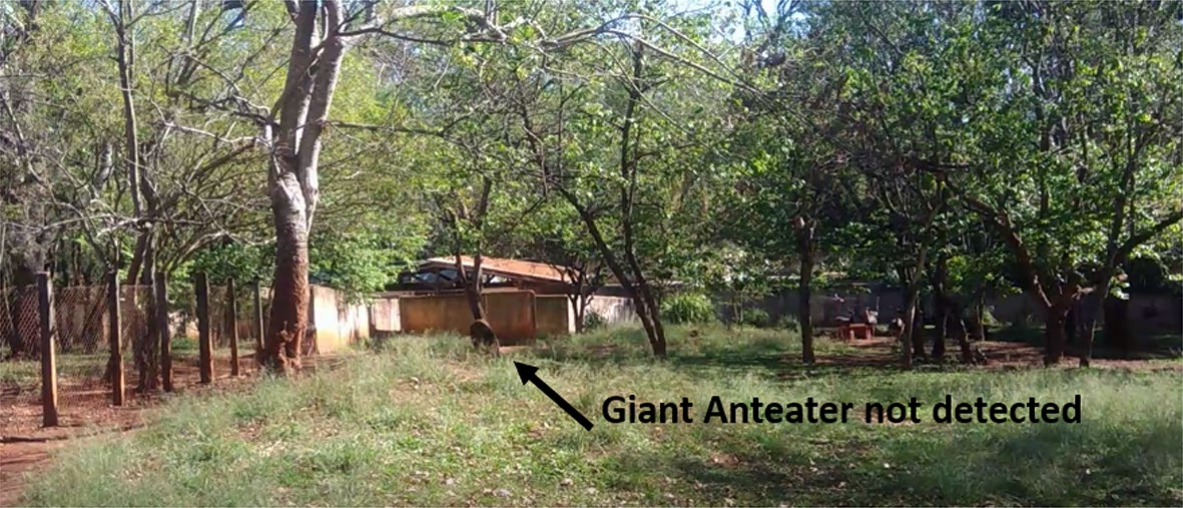
An example of the camouflage and small object challenge in the Giant Anteater class, in which the animal is in the same color range as the vegetation and at a distance away from the data input, making detection difficult.

In this work two different types of videos were used, the first set of videos consists of videos related to the computer vision challenges described, each video lasts from 30 to 45 s and there was only the possibility of obtaining the challenges with two classes (Fig. 17 Tapir class and Fig. 18 Giant Anteater class), due to the complexity of its enclosures being open and enabling the creation of situations of occlusion, small objects, and camouflage. The second set of videos was played for each class, which does not have the challenges, in which each video is 20–30 s and contained a single animal, in addition, the recording camera angle was 45°, an example of a clean video can be seen in Fig. 16.

Among the models, the more complex ones were almost able to handle the problem of occlusion. However, none of the models were able to detect small animals, likely due to a lack of examples in the dataset. In terms of camouflage, none of the models were successful. The ability of an animal to camouflage itself or its involuntary camouflage due to a similar coloration to its surroundings can result in many false positives, detecting animals that are not present.

## Conclusion

This article aimed to compare various YOLO architectures to identify those that can be used to build animal detection systems on highways. Models were trained with and without data augmentation to determine which models struggled with the small domain dataset even after augmentation.

In tests on the validation set, the Scaled-YoloV4 model achieved the best results in mitigating false negatives (better recall), showing greater efficiency for detecting endangered Brazilian animals with the lowest percentage of false negatives. The Scaled-YoloV4 model also had the highest accuracy, while the YoloV4 model had the highest mAP@50. In terms of average FPS on video inference, the YoloV5-N model was the most performant. It can be concluded that the YoloV4, Scaled-YoloV4, YoloV5, and YoloR architectures provide relevant performances for creating real-time animal detection systems and that data augmentation techniques are effective and efficient for training these architectures even with a limited domain dataset. In total, we concluded that the application of data augmentation techniques was effective, with all models having at least one metric improved after application (exception to YoloV7). In comparison, the YoloV7 and YoloX detectors had lower-than-expected results on the validation set, due to their specialized convolution filters for higher image resolutions being less efficient on inputs smaller than HD. In terms of FPS, the decoupled architecture of the YoloX models drastically reduced performance, while the YoloV7 model maintained high speed even with large and complex versions. It is also possible to conclude that the use of architectures on edge devices with low RAM space is still a challenge for large and complex networks, even in recent YOLO versions. Unlike the performance of the models on the GPU, the inference execution speed of the models is still very low.

However, scenarios with classic computer vision problems still represent challenges that must be considered for animal detection. It can be inferred that all architectures may face difficulties with small datasets that have limited variation in animal poses, few distant (small) animals, or non-standard image dimensions, as is the case with the BRA-Dataset. These challenges may also apply to other animal detection scenarios around the world.

For future work, we plan to reassess the BRA-Dataset and consider possible additions and improvements. We will also explore new emerging data augmentation techniques for future comparisons, like techniques to simulate unfavorable scenarios for detection in order to provide a larger sample of images for training. Further contributions include implementing other single-stage detection architectures for comparison with those based on YOLO, which could expand our understanding of the challenges of animal detection. Testing YOLO architectures on specialized artificial intelligence devices for edge computing (e.g Nvidia Jetson Family, FPGA devices and others) could provide valuable insights into the practical application of remote real-time detection and help evaluate memory and processing consumption. Additionally, evaluating the models in other occlusion scenarios could help address animal detection challenges in different environments around the world and provide other researchers with a better understanding of the technologies available in their local environment.

## Data Availability

The publication titled “Brazilian Road’s Animals (BRA): An Image Dataset of Most Commonly Run Over Animals” can be accessed with the 10.1109/SIBGRAPI55357.2022.9991774. The associated research data can be found at the website address https://github.com/GabrielFerrante/BRA-Dataset. This repository contains the dataset utilized in the publication, specifically curated for the purpose of studying commonly run over animals on Brazilian roads, along with the provided links to access the corresponding images. In addition, the repository for the experiments can be accessed via the following link: https://github.com/GabrielFerrante/DetectAnimalsInRoads.
